# Evidence of space–time clustering of childhood acute lymphoblastic leukaemia in Sweden

**DOI:** 10.1038/sj.bjc.6690103

**Published:** 1999-02

**Authors:** B Gustafsson, J Carstensen

**Affiliations:** Department of Paediatrics, Huddinge Hospital, Karolinska Institute, Huddinge, 141 86, Sweden; Oncologic Centre, University Hospital, Linköping, S-581 85, Sweden; Department of Health and Society, University of Linköping, Linköping, S-581 83, Sweden

**Keywords:** childhood acute lymphatic leukaemia, space–time clustering, viral hypothesis

## Abstract

We have examined 645 recorded cases of childhood acute lymphatic leukaemia (ALL) in Sweden during 1973–89 to identify space–time clustering by using the close-pair method of Knox. The records included date of birth and of diagnosis as well as addresses at birth and at diagnosis. There was a significant excess of case pairs close in date of birth and place of birth in the 5- to 15-year age group. © 1999 Cancer Research Campaign


*British Journal of Cancer* (1999) **79**(3/4), 655–657
© 1999 Cancer Research Campaign
Article no. bjoc.1998.0103

Short communication

### Evidence of space–time clustering of childhood acute lymphoblastic leukaemia in Sweden

**B Gustafsson[1] and J Carstensen[2,3]**

[1]Department of Paediatrics, Huddinge Hospital, Karolinska Institute, 141 86 Huddinge, Sweden; [2]Oncologic Centre, University Hospital, S-581 85 Linköping, Sweden; [3]Department of Health and Society, University of Linköping, S-581 83 Linköping, Sweden

**Summary** We have examined 645 recorded cases of childhood acute lymphatic leukaemia (ALL) in Sweden during 1973–89 to identify space–time clustering by using the close-pair method of Knox. The records included date of birth and of diagnosis as well as addresses at birth and at diagnosis. There was a significant excess of case pairs close in date of birth and place of birth in the 5- to 15-year age group.

**Keywords:** childhood acute lymphatic leukaemia; space–time clustering; viral hypothesis

Established aetiological factors for childhood leukaemia including genetic predisposition, exposure to moderate/high doses of ionizing radiation and chemotherapeutic agents explain relatively few cases (Doll, 1989). The only known infectious cause of leukaemia is the retrovirus human T-lymphotropic virus type 1 (HTLV 1), which causes adult T-cell leukaemia/lymphoma. HTLV-1 infection clusters geographically, but is rare outside the Caribbean, equatorial Africa and southern Japan (Tajima, 1990).

Early suspicions of the existence of an infectious agent in childhood leukaemia relied to a great extent on reports of clusters of new cases localized in space and time, although the validity of these findings has been questioned (Smith, 1982). Recently, results of a series of large space–time studies in Britain (Gilman and Knox, 1991; Alexander, 1992; Knox and Gilman, 1992; Gilman and Knox, 1995) and Greece (Petridou et al, 1996) have strengthened the evidence for the existence of space–time clustering. The British studies indicate clustering not only at diagnosis but also at or around birth, however, at both times the effect applies to only a small proportion of cases. (Alexander, 1992; Gilman and Knox, 1995).

Furthermore, the results from both earlier (Knox, 1964) and more recent studies of space–time clustering (Alexander, 1992; Petridou et al, 1996) suggest differences between younger and older children. Alexander (1992) found strong indirect confirmation of a prior hypothesis that ‘some children become persistently infected followed exposure in utero or around the time of birth. These children have an increased risk of developing leukaemia, especially ALL at older age of onset (5 years or older)’. This analysis showed weaker evidence to support the theory that a recent infection may contribute to acute lymphatic leukaemia (ALL) in the childhood peak (2–4 years of age).

We have studied childhood leukaemia records from Sweden to identify space–time clustering in space and time of birth and at diagnosis. We have linked the Swedish Cancer Register to the Swedish Medical Birth Register, which contains information on nearly all infants born in Sweden since 1973 (Cnattingius and Ericson, 1990). Data were then analysed using the close-pair method of Knox.

*Received 26 February 1998*
*Revised 8 July 1998*
*Accepted 13 July 1998*

*Correspondence to:* B Gustafsson

### MATERIAL AND METHODS

Six hundred and nine children with acute lymphatic leukaemia (ICD 204.0) and 36 with acute unspecified leukaemia (ICD 207.0) born and diagnosed between 1973 and 1989 in Sweden in the 0–15 year age group at diagnosis comprised the study material. Information on age and address at diagnosis of the cases was obtained from the Swedish Cancer Register, which is a compulsory register of cancers including practically all cases of malignancies during this period (Mattson, 1984). We obtained the addresses at birth from the Swedish Medical Birth Register, which is a standardized set of medical records introduced in Sweden in 1973 (Cnattingius and Ericson, 1990).

Knox’s method for detecting space-time clustering was used for the statistical analysis (Knox, 1963). With the Knox approach, two cases are considered to be close neighbours if they reside in both a specified spatial and a specified temporal distance of each other. The possible pairs considered are $n$ ($n$–1)/2 pairs for $n$ observed cases of leukaemia. Five critical intervals between dates of diagnosis (or birth) were used: 1, 3, 6, 12 and 24 months. Pairs of cases diagnosed (or born) within the same municipality were considered as close in space. We used the definition of municipalities from 1977, which divides Sweden into 277 municipalities. The mean number of inhabitants was 30 000 (range 4 000–700 000) and the mean area was 1486 km$^2$ (range 9–19 447 km$^2$). The statistical significance was determined assuming Poisson variation.

### RESULTS

The results of our analyses indicate significant clustered pairs of children according to municipality at birth and the date of birth in cases aged 5 years old or more (Table 1). The most striking feature was an increased relative risk for pairs residing within the same municipality and born within 1 month of each other, 11 observed close pairs against an expected value of 4.4 ($P = 0.006$). Even after accounting for multiple testing using Boneferroni’s rule (by multiplying this $P$-value with the number of critical time intervals

**Table 1** Observed and expected numbers of pairs of cases of acute lymphatic leukaemias within the same municipality and the same time interval among Swedish children, 1973–89

| | Critical time intervals (months) | | | | |
|---|---|---|---|---|---|
| **Age at diagnosis** | **1** | **3** | **6** | **12** | **24** |
| Linkage according to municipality at birth and date of birth | | | | | |
| 0–15 years | | | | | |
| Observed | 41 | 94 | 196 | 375 | 704 |
| Expected | 33.4 | 100.1 | 196.9 | 385.6 | 743.5 |
| Obs./exp. | 1.23 | 0.94 | 1.00 | 0.97 | 0.95 |
| 0–4 years | | | | | |
| Observed | 18 | 43 | 93 | 192 | 346 |
| Expected | 15.5 | 47.5 | 93.2 | 184.6 | 355.5 |
| Obs./exp. | 1.16 | 0.91 | 1.00 | 1.04 | 0.97 |
| 5–15 years | | | | | |
| Observed | 11 | 18 | 33 | 50 | 98 |
| Expected | 4.4 | 12.2 | 24.4 | 47.3 | 91.0 |
| Obs./exp. | 2.50* | 1.47 | 1.35 | 1.06 | 1.08 |
| Linkage according to municipality at diagnosis and date of diagnosis | | | | | |
| 0–15 years | | | | | |
| Observed | 32 | 82 | 170 | 345 | 649 |
| Expected | 31.2 | 88.5 | 171.0 | 333.7 | 641.2 |
| Obs./exp. | 1.04 | 0.93 | 0.99 | 1.03 | 1.01 |
| 0–4 years | | | | | |
| Observed | 15 | 37 | 82 | 167 | 318 |
| Expected | 14.2 | 41.2 | 79.5 | 155.8 | 301.8 |
| Obs./exp. | 1.05 | 0.90 | 1.03 | 1.07 | 1.05 |
| 5–15 years | | | | | |
| Observed | 1 | 7 | 20 | 37 | 64 |
| Expected | 3.5 | 10.3 | 19.7 | 37.6 | 72.0 |
| Obs./exp. | 0.28 | 0.68 | 1.01 | 0.98 | 0.89 |

*$P = 0.006$ (one-sided).

tested), there was still a clear significance for clustering (adjusted $P = 5 \times 0.006 = 0.030$). The observed close pairs involved 19 cases, and includes one larger space–time cluster in the middle part of Sweden with three cases. The cases are spread all over the country. If we exclude the cases of acute unspecified leukaemia (36), we find an even more significant result because none of these cases belonged to the clusters (observed number of case pairs = 11, expected number = 3.7, $P = 0.002$).

In contrast, neither among children aged 0–4 years nor among children aged 0–15 years was there any significant space–time clustering (Table 1). Linkage according to municipality at diagnosis and date of diagnosis among children 0–4 years, 5–15 years and 0–15 years of age showed no significant excess of case pairs.

### DISCUSSION

Clustering of leukaemia would be of appreciable interest because it would add weight to the view that some childhood leukaemias have a viral origin (Alexander, 1993). Recent evidence has emerged of clustering in time and space of both birth and diagnosis, indicating that the latent period between initiation and recognition of cancer is variable and can be long (Gilman and Knox, 1995). The ‘striking peak’ of ALL around 3–5 years of age might have a different aetiological agent from ALL with onset after the childhood peak. Although most studies indicate evidence of clustering in the youngest age groups (Smith, 1982; Alexander, 1993; Kinlen, 1995), we refer here to the spatial–temporal pattern of clustering reported by Alexander (1992). She suggests that some children develop a persistent infection after exposure in utero or around the time of birth. These children run an increased risk of developing leukaemia, especially at an older age of onset. Results of this analysis suggest that transmission of this specific, though unknown, agent plays some role in the development of childhood acute lymphoblastic leukaemia at the times when children are susceptible to infection. The hypothetical latent period between exposure and leukaemia is particularly long for this group. Weaker evidence was provided to support the theory of a more recent infectious exposure that may contribute to ALL in the childhood peak years (Alexander, 1992).

The space–time analysis has recently been replicated in the ‘EUROCLUS’ project including residence at diagnosis for 13 351 cases of childhood leukaemia diagnosed during 1980–89 in defined geographical regions in 17 countries. The results indicate statistically significant evidence of clustering of ALL in the childhood peak years as well as ALL at older age of onset, although the magnitude is small (Alexander et al, 1998).

Kinlen and co-workers have reported that population mixing can raise the incidence of leukaemia by facilitating transmission of the postulated underlying infective agent(s) (Kinlen, 1988, 1995; Kinlen et al, 1990). Immigration to rural areas, particularly if remote or isolated, offers opportunities for spread of viral infections, to which most urban populations become relatively immune

 *© Cancer Research Campaign 1999*

at a very early age. Recent studies from Hong Kong and Greece have further confirmed this hypothesis (Petridou et al, 1996; Alexander et al, 1997).

Our aim in this study was to apply the theory of Alexander and the method of Knox to a Swedish community. We exploited the unique situation of a Cancer Register that could be linked to the Medical Birth Register, both registers that are well documented as to being accurate (Mattson, 1984; Cnattingius and Ericson, 1990).

This present study is the first in Sweden that gives evidence of a birth date and space–time clustering of children who later developed acute lymphatic leukaemia. Such a phenomenon would support the hypothesis that infections in early life can increase the risk. However, it should be pointed out that only a small portion of the cases are involved in the clusters. In conjunction with other evidence, this indicates that childhood leukaemia could be an uncommon response to the relevant infection, but further studies are needed to establish this.

### REFERENCES

Alexander FE (1992) Space–time clustering of childhood acute lymphoblastic leukaemia: indirect evidence for a transmissible agent. *Br J Cancer* **65**: 589–592

Alexander FE (1993) Viruses, clusters and clustering of childhood leukaemia, a new perspective? *Eur J Cancer* **29**: 1424–1443

Alexander FE, Chan LC, Lam TH, Yuen P, Leung NK, Ha SY, Yuen HL, Li CK, Lau YL and Greaves MF (1997) Clustering of childhood leukaemia in Hong Kong: association with the childhood peak and common acute leukaemia and with population-mixing. *Br J Cancer* **75**: 457–463

Alexander FE, Boyle P, Carli P-M, Coebergh JW, Draper GJ, Ekbom A, Levi F, McKinney PA, McWhirter W, Magnanai C, Michaelis J, Olsen JH, Peris-Bonet R, Petridou E, Pukkala E and Vatten L (1998) Spatial temporal patterns in childhood leukaemia: further evidence of an infectious origin. *Br J Cancer* **77**: 812–817

Cnattingius S and Ericson A (1990) A quality of study of a medical birth registry. *Scand J Soc Med* **18**: 143–148

Doll R (1989) The epidemiology of childhood leukaemia. *J R Stat Soc Series* **152**: 341–351

Gilman EA and Knox EG (1991) Temporal–spatial distribution of childhood leukaemias and non-Hodgkin lymphomas in Great Britain. In *The Geographical Epidemiology of Childhood Leukaemia and Non-Hodgkin Lymphomas in Great Britain, 1966–1983*. Draper GJ (ed.). Studies on Medical and Population Subjects No 53. HMSO: London

Gilman EA and Knox EG (1995) Childhood cancers: space–time distribution in Britain. *J Epidemiol Community Health* **49**: 158–163

Kinlen LJ (1988) Evidence for an infective cause for childhood leukaemia comparison of a Scottish new town with nuclear reprocessing sites in Britain. *Lancet* **ii**: 1323–1327

Kinlen LJ (1995) Epidemiological evidence for an infective basis in childhood leukaemia. *Br J Cancer* **71**: 1–5

Kinlen LJ, Clarke K and Hudson C (1990) Evidence from population-mixing in British new towns, 1946–1985, of an infective basis of childhood leukaemia. *Lancet* **336**: 577–582

Knox EG (1963) The detection of space–time interactions. *Appl Stat* **13**: 25–29

Knox EG (1964) Epidemiology of childhood leukaemia in Northumberland and Durham. *Br J Prev Soc Med* **18**: 17–24

Knox EG and Gillman E (1992) Leukaemia clusters in Great Britain. 1. Space–time interactions. *J Epidemiol Community Health* **46**: 566–572

Mattson B (1984) *Cancer Registration in Sweden*. Department of oncology and cancer epidemiology. Karolinska Hospital: Stockholm

Petridou E, Revinthi K, Alexander FE, Haidas S, Koliouskas D, Kosmidis H, Piperopoulou F, Tzortzatou F and Trichopoulos D (1996) Space–time clustering of childhood leukaemia in Greece: evidence supporting a viral aetiology. *Br J Cancer* **73**: 1278–1283

Smith PG (1982) Spatial and temporal clustering. In *Cancer Epidemiology and Prevention*. Scholtenfeld D and Fraumeni JF (eds.), pp. 391–407. Saunders: Philadelphia

Tajima K and the T- and B-cell Malignancy Study Group (1990) The 4th nationwide study of adult T-cell leukemia/lymphoma (ATL) in Japan: estimates risk of ATL and its geographical distribution and clinical features. *Int J Cancer* **45**: 237–243

*© Cancer Research Campaign 1999*

